# Exploratory analysis of poultry workers’ knowledge and practices Regarding highly pathogenic avian influenza in Guinea

**DOI:** 10.1371/journal.pone.0320890

**Published:** 2025-03-27

**Authors:** Maladho Diaby, Salifou Talassone Bangoura, Castro Gbêmêmali Hounmenou, Kadio Jean-Jacques Olivier Kadio, Aly Badara Touré, Kouramoudou Bereté, Emile Faya Bongono, Sidikiba Sidibé, Alexendre Delamou, Alioune Camara, Alpha-Kabinet Keita, Abdoulaye Touré

**Affiliations:** 1 Guinea Infectious Diseases Research and Training Center (CERFIG), Gamal Abdel Nasser University, Conakry, Republic of Guinea; 2 Department of Pharmaceutical and Biological Sciences, Gamal Abdel Nasser University, Conakry, Republic of Guinea; 3 Department of Public Health, Gamal Abdel Nasser University, Conakry, Republic of Guinea; 4 African Centre of Excellence in the Prevention and Control of Communicable Diseases (CEA-PC MT), Faculty of Sciences and Health Techniques, Gamal Abdel Nasser University, Conakry, Republic of Guinea; 5 Department of the Central Veterinary Laboratory of Guinea (LCVD), Ministry of Agriculture and Livestock, Conakry, Republic of Guinea; 6 TransVIHMI, IRD/INSERM/Monpellier University, Montpellier, France; University of Ibadan Faculty of Veterinary Medicine, NIGERIA

## Abstract

**Background:**

In 2022-2023, Guinea experienced a major avian influenza epizootic, leading to significant economic losses and increasing the risk of transmission to humans. Raising awareness and promoting protective behaviour among the general population, particularly high-risk groups, could help strengthen prevention and control measures for this zoonosis. This study aimed to assess knowledge and practices related to avian influenza among poultry workers in Guinea.

**Methods:**

A cross-sectional study was conducted between November and December 2023 on poultry farms in Coyah and Forecariah, Guinea prefectures. A survey was administered to all poultry farms in these two prefectures. Data were collected using a questionnaire, which included the following variables: socio-demographic and professional profile of respondents, avian influenza information, sources of information, and a series of questions to assess their knowledge and practices. Knowledge and practice scores were then calculated. The cumulative local effects method was used to assess the influence and contribution of each co-variate to changes in the probability of knowledge and practice levels among poultry farm staff.

**Results:**

A total of 326 poultry workers participated in the survey, including poulterers (62.3%), managers (17.5%), and poultry technicians (13.8%). More than half of these workers (54.9%) had heard of influenza avian. Their primary sources of information were health workers (27.9%), friends and fellow farmers (23.3%) and employees (22.7%). Overall, the knowledge of avian influenza was relatively low among poultry workers (42.9%), and the majority (68.4%) demonstrated poor practices on poultry farms. Analysis using the ALE model reveals that age, education and type of occupation are significantly associated with knowledge. At the same time, the number of farms managed, the number of hours worked, and gender are associated considerably with practices among these workers.

**Conclusion:**

The study revealed a low level of knowledge and poor practices among poultry farm workers despite an avian flu epizootic. These findings suggest the need for a targeted strategy to prevent the risk of virus transmission to humans, including awareness-raising, training, and providing personal protective equipment.

## Introduction

Previously known as fowl plague, highly pathogenic avian influenza (HPAI) or bird flu is a potentially zoonotic, highly contagious viral infection that affects wild birds and poultry. It can be transmitted to humans, primarily causing respiratory infections, and may also affect other warm-blooded animals, making it an emerging zoonotic pandemic [1]. Subtypes H5 and H7 are major pathogens with high morbidity and mortality, leading to substantial losses in the global poultry industry and posing significant public health and food safety risks [2–4].

Highly pathogenic avian influenza has affected numerous countries and territories worldwide, with occasional cases of human infection caused by the H5N1 subtype [5]. According to the World Health Organization, 954 cases of human infection with influenza A(H5N1) were recorded in 24 countries between 2003 and 2024, resulting in 464 deaths [6]. Consequently, the disease continues to pose a growing pandemic threat [7].

In Africa, 361 cases of human infection, resulting in 121 deaths, were reported in three countries between 2006 and 2017: Egypt (359 cases and 120 deaths), Nigeria (1 case and 1 death) and Djibouti (1 case and no deaths) [6]. In other countries such as Burkina Faso, Côte d’Ivoire, Ghana, Niger, Cameroon, and Togo, cases of H5N1 infection have been observed in poultry, but no human infection has been reported [8,9]. In Guinea, after several suspicions in previous years, the Ministry of Agriculture and Livestock officially declared the first epizootic of highly pathogenic avian influenza of subtype H5N1 in June 2022, affecting Coyah and Forecariah prefectures, suburbs of the capital Conakry [10]. Over a hundred farms were impacted by the H5N1 infection, with an average poultry mortality rate estimated at around 40% [11]. No human infection by the H5N1 virus has been reported.

The majority of human infections with the virus have been linked to the direct handling and consumption of infected poultry and poultry products, as well as visits to live poultry markets [3,12,13]. Contact with contaminated environments, such as water or poultry droppings used as fertilizer or fish feed, could also pose an infection risk for people without direct exposure to infected poultry [3,14]. Thus, employees in the poultry industry and on poultry farms who are frequently in contact with sick or dying poultry represent a high-risk group that preventive health education programs should target. Their elevated exposure is due to their handling and preparation of raw poultry meat and products [13]. Without data indicating an avian influenza epidemic in the human population, it is essential to assess the knowledge and practices surrounding this public health issue, particularly among those most at risk. This study aimed to assess the knowledge and practices of poultry workers in the prefectures of Coyah and Forecariah during the avian influenza epidemic in Guinea.

## Materials and methods

### Sitting and study design

**Fig 1:**
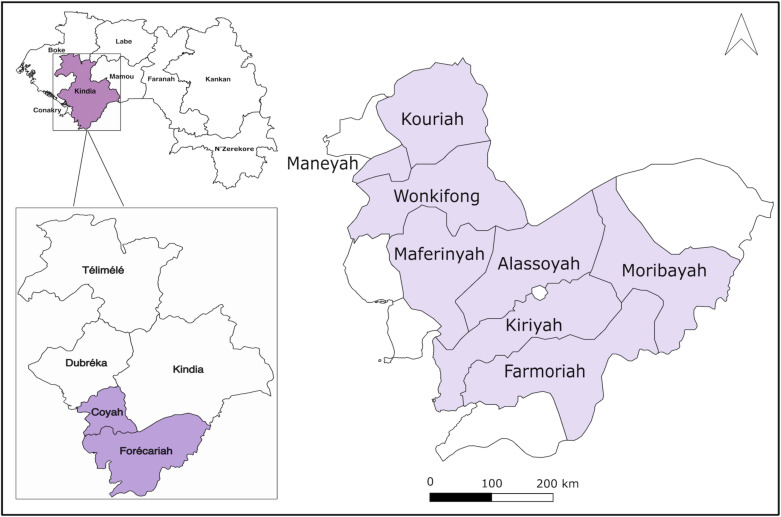
Map of districts affected by avian influenza in the Coyah and Forecariah prefectures.

A cross-sectional survey was conducted between November and December 2023 among the staff of poultry farms in the prefectures of Coyah and Forecariah, which was the epicentre of the 2022-2023 epizootic (Fig 1). The poultry industry is a significant economic activity in these two prefectures, generating substantial income [15]. These areas were selected due to the large number of poultry farms and the previous H5N1 avian influenza outbreak.

### Population and data collection

We included all workers who were present on the farms and who agreed to participate in the study. They included veterinarians, poultry breeders, egg collectors, managers, animal feed managers and farm workers. Data were collected using a standardized questionnaire based on the WHO fact sheet on avian influenza [16] and a study of workers in the Italian poultry industry [17]. The questionnaire was administered face-to-face to the participants. The data collected included socio-demographic and occupational characteristics such as age, sex, education, role or occupation on the farm (farm owner or employee), general knowledge of avian influenza, and prevention practices. Additional questions assessed the source of information. Respondents’ knowledge was evaluated using 35 questions on the definition, modes of transmission, and prevention measures for H5N1 avian influenza. Each correct answer was scored ‘1’, while incorrect answers or “don’t know” were scored ‘0’. Scores were summed and categorized according to modified Bloom’s Taxonomy criteria [18]: good knowledge if the score was ≥ 70%, otherwise poor knowledge. Participants’ practices were assessed using eight questions on preventive measures, with responses rated on a scale of 1 to 5 (never, rarely, sometimes, often, always). The practice score was calculated by summing responses and categorized as good practice if the score was ≥  70% of the total (40 points); otherwise, practices were classified as poor.

### Statistical analysis

Categorical variables were summarized as percentages, while quantitative variables were described using median and interquartile ranges. Bivariate statistical analyses (Chi-square test or Fisher’s exact test) were conducted to compare the proportions of poultry farm workers’ knowledge levels regarding the definition, transmission modes, at-risk professional groups, and avian influenza prevention measures, as well as their practice levels on poultry farms, in relation to socio-demographic and professional characteristics (p-value <  0.05 indicating significant association).

Additive generalized linear models, GAM (a special case of supervised learning models of multilayer perceptron-type neural networks under certain conditions [19] were used to identify the determinants of good knowledge and good practices of poultry farm workers concerning their socio-demographic characteristics. The agnostic model follows them, cumulative local effects [20,21], whose outputs are in graphical form to facilitate the interpretation of these determinants. The implementation steps of this hybrid multivariate approach are: (i) Subdivision of each dataset, 70% as training data and 30% as test data; (ii) application of the Random Over-Sampling (ROSE) technique to balance the initially unbalanced binary classes with the function “ovun. sample’‘ function of the “ROSE’‘ package; (iii) implementation of GAM with flexible curves on the training data using the “gam’‘ function of the “mgcv’‘ package and determination of the best model that best fits the test data based on the lowest Akaike Information Criterion (AIC) and finally (iv) implementation of ALE on the test data with the “ale’‘ function of the “ale’‘ package. R 4.2.2 was used to process and analyze the data. The map was created with QGIS software (source of administrative boundaries map layer: https://gadm.org/, link to the GADM license: https://gadm.org/license.html).

### Ethical considerations

The National Health Research Ethics Committee approved the protocol (088/CNERS/23). An information note explained the study’s objectives and expected outcomes. No personal identifying information was collected to ensure anonymity and confidentiality; only the principal investigator had access to the data. Each participant provided voluntary written consent before participating in the study.

## Results

### Socio-demographic characteristics of farm workers

The Prefecture Livestock Directorates of Coyah and Forecariah surveyed 224 poultry farms, of which 107 were reportedly infected with the highly pathogenic H5N1 avian influenza virus.

This study included 122 farms, with 326 individuals participating in the survey, 193 of whom worked on poultry farms in Coyah and 133 in Forecariah. Participants were predominantly male (93.9%) and young, with a median age of 30 years (IQR: 25-38). More than half (58%) of the respondents were married, and 63.8% had attended school.

Over two-thirds (68.7%) of respondents had been involved in poultry farming for less than five years. Participants were primarily poultry farmers (62.3%), managers (17.5%), poultry technicians (13.8%) and veterinarians (4.6%). Over half of the participants (54.9%) had received information about avian influenza, 40.8% of whom received it several months ago and 58.7% several years ago. The primary sources of information were healthcare professionals (27.9%), colleagues and friends (23.3%), employers (22.7%) and the media (19%). Additionally, the vast majority of participants (92.9%) expressed a need for information on avian influenza, particularly regarding preventive measures (70.9%), epidemiology (69.6%), modes of transmission (54.9%), clinical features (40.2%) and case management (39%) (Table 1).

**Table 1:** Socio-demographic and occupational features and level of information of workers on H5N1 avian influenza infection in Coyah and Forecariah prefectures, 2023.

Characteristics	Overall
	N = 326 (%)
**Gender**	
Male	306 (93.9)
Female	20 (6.1)
**Age (years)**	
<25	78 (23.9)
25–30	98 (30.1)
31–40	89 (27.3)
>40	61 (18.7)
**Marital status**	
Unmarried	137 (42.0)
Married	189 (58.0)
**Education**	
Unschooled	118 (36.2)
Schooled	208 (63.8)
**Occupation on the farm**	
Veterinarian	15 (4.6)
Poulterer	203 (62.3)
Poultry technician	45 (13.8)
Manager	57 (17.5)
Support functions	6 (1.8)
**Year of experience**	
<5 years	224 (68.7)
5 years and over	102 (31.3)
**Farm status**	
Infected farm	104 (31.9)
Uninfected farm	222 (68.1)
**Information about influenza avian received previously**	
No	147 (45.1)
Yes	179 (54.9)
**When this information on influenza avian was received**	
3 to 4 weeks	1 (0.6)
Several months ago	73 (40.8)
Several years ago	105 (58.7)
**Sources of information**	
Medias	62 (19.0)
Employer	74 (22.7)
Internet/Social networks	18 (5.5)
Magazines	1 (0.3)
Friends/Colleagues/Family	76 (23.3)
Healthcare workers	91 (27.9)
Ministry/ Livestock Department	13 (4.0)
**Information needs on influenza avian**	
No	23 (7.1)
Yes	303 (92.9)
**Types/categories of needed information**	
Epidemiology	227 (69.6)
Prevention measures	231 (70.9)
Mode of transmission	179 (54.9)
Clinical characteristics	131 (40.2)
Management	127 (39.0)

### Knowledge and practices of poultry workers about H5N1 avian influenza

#### Overall knowledge.

**Fig 2:**
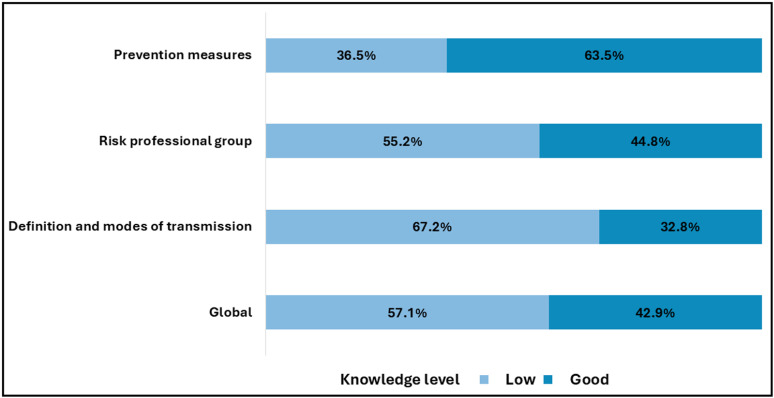
Poultry workers’ overall knowledge regarding the definition and modes of transmission, at-risk professional groups, and preventive measures for avian influenza.

More than half of the participants (57.1%) had a low level of knowledge about avian influenza. Most respondents were unaware of how to define H5N1 avian influenza or its modes of transmission (67.2%). Conversely, over half of the respondents (55.2%) had a good understanding of the occupational groups at risk of contracting the avian flu virus, and 63.5% were knowledgeable about preventive measures (Fig 2).

### Knowledge of the definition and modes of transmission

The participants’ knowledge of the definition and modes of transmission of H5N1 avian influenza varied by age group (p < 0.004). Those over 30 years demonstrated good knowledge of the definition and transmission methods than younger participants. Likewise, respondents who had attended school (41.3%) exhibited a higher level of knowledge regarding the definition and transmission methods of H5N1 avian influenza than those without formal education (17.8%) (p <  0.001). Among farm workers, veterinarians and managers had superior knowledge of the definition and transmission methods compared to their peers in other roles (p <  0.001) (Table 2).

**Table 2:** Poultry workers’ knowledge of the definition and modes of transmission, professionals at risk, and avian influenza prevention measures.

Characteristic	Definition and modes of transmission			Risk professional group			Prevention measures		
	Low n = 219 (%)	Good n = 107 (%)	*p*	Low n = 180 (%)	Good n = 146 (%)	*p*	Low n = 119 (%)	Good n = 207 (%)	*p*
**Gender**			0.4			0.6			0.9
Male	204 (66.7)	102 (33.3)		170 (55.6)	136 (44.4)		112 (36.6)	194 (63.4)	
Female	15 (75.0)	5 (25.0)		10 (50.0)	10 (50.0)		7 (35.0)	13 (65.0)	
**Age (years)**			0.004			0.003			<0.001
<25	64 (82.1)	14 (17.9)		57 (73.1)	21 (26.9)		46 (59.0)	32 (41.0)	
25–30	67 (68.4)	31 (31.6)		52 (53.1)	46 (46.9)		33 (33.7)	65 (66.3)	
31–40	50 (56.2)	39 (43.8)		41 (46.1)	48 (53.9)		19 (21.3)	70 (78.7)	
>40	38 (62.3)	23 (37.7)		30 (49.2)	31 (50.8)		21 (34.4)	40 (65.6)	
**Education**			<0.001			<0.001			<0.001
Unschooled	97 (82.2)	21 (17.8)		86 (72.9)	32 (27.1)		60 (50.8)	58 (49.2)	
Schooled	122 (58.7)	86 (41.3)		94 (45.2)	114 (54.8)		59 (28.4)	149 (71.6)	
**Occupation on the farm**			<0.001			<0.001			<0.001
Veterinarian	6 (40.0)	9 (60.0)		3 (20.0)	12 (80.0)		1 (6.7)	14 (93.3)	
Poulterer	153 (75.4)	50 (24.6)		128 (63.1)	75 (36.9)		88 (43.3)	115 (56.7)	
Poultry technician	40 (88.9)	5 (11.1)		32 (71.1)	13 (28.9)		18 (40.0)	27 (60.0)	
Manager	14 (24.6)	43 (75.4)		11 (19.3)	46 (80.7)		10 (17.5)	47 (82.5)	
Support functions	6 (100.0)	0 (0.0)		6 (100.0)	0 (0.0)		2 (33.3)	4 (66.7)	
**Year of experience**			0.10			0.13			0.4
<5 years	157 (70.1)	67 (29.9)		130 (58.0)	94 (42.0)		85 (37.9)	139 (62.1)	
5 years and over	62 (60.8)	40 (39.2)		50 (49.0)	52 (51.0)		34 (33.3)	68 (66.7)	
**Farm status**			0.8			0.7			0.082
Infected farm	69 (66.3)	35 (33.7)		56 (53.8)	48 (46.2)		45 (43.3)	59 (56.7)	
Uninfected farm	150 (67.6)	72 (32.4)		124 (55.9)	98 (44.1)		74 (33.3)	148 (66.7)	

### Knowledge of professional groups at risk

Regarding knowledge of at-risk professional groups, over 50% of participants aged 31-40 and those over 40 demonstrated good awareness of at-risk groups. Conversely, participants aged under 25 exhibited low knowledge of these groups. The difference between age categories was statistically significant (p < 0.003). Additionally, the level of knowledge regarding occupational groups at risk varied significantly based on the education and occupation of the participants on the farm (p < 0.001). Approximately 55% of participants who attended school had a good understanding of the occupational groups at risk of H5N1 avian influenza infection, compared to 27.1% of those who did not attend school. Moreover, more than two-thirds of veterinarians and managers showed a good understanding of the occupational groups at risk. At the same time, poultry farmers (36.9%) and poultry technicians (28.9%) demonstrated lower levels of knowledge (Table 2).

### Knowledge of prevention measures

Knowledge of prevention measures for H5N1 avian influenza infection was significantly associated with participants’ age, education, and occupation on the farm (p < 0.001). Participants aged 25 years and older demonstrated a good understanding of prevention measures compared to those under 25. Approximately 72% of participants who had attended school displayed a good knowledge of H5N1 avian influenza infection prevention measures, compared to 49.2% of those who had not. Furthermore, participants exhibited good knowledge of preventive measures regardless of their occupation on the farm, with higher proportions among veterinarians (93.3%) and farm managers (82.5%) (Table 2).

### Practices

Our results indicated that good practices were observed as the number of hours worked increased (p < 0.001). However, poor practices were prevalent among the younger age groups, particularly those under 30. Participants who had attended school also recorded higher poor practice scores (68.8%) than those who had not. Furthermore, regardless of the worker’s occupation on the farm, the poor practice scores were nearly identical across all groups (p > 0.9).

Workers with fewer years of experience recorded the highest poor practice score (68.8%) compared to those with more experience. Conversely, individuals working on farms infected with avian influenza exhibited a higher score for good practices (37.5%) than those on non-infected farms (28.8%) (Table 3).

**Table 3:** Practices of poultry workers on farms in Coyah and Forecariah, 2023.

Characteristic	Practice		p-value
	Poor Practice	Good practice	
	N = 223	N = 103	
**Usual number of farm**			0.8
>1	11 (73.3%)	4 (26.7%)	
1	212 (68.2%)	99 (31.8%)	
**Number of hours worked**	10.00 (9.00, 12.00)	11.00 (10.00, 12.00)	<0.001
**Gender**			0.032
Male	205 (67.0%)	101 (33.0%)	
Female	18 (90.0%)	2 (10.0%)	
**Age (years)**			0.4
<25	57 (73.1%)	21 (26.9%)	
25–30	70 (71.4%)	28 (28.6%)	
31–40	55 (61.8%)	34 (38.2%)	
>40	41 (67.2%)	20 (32.8%)	
**Education**			0.9
Unschooled	80 (67.8%)	38 (32.2%)	
Schooled	143 (68.8%)	65 (31.3%)	
**Number of years worked**			0.8
<5 years	154 (68.8%)	70 (31.3%)	
5 years and over	69 (67.6%)	33 (32.4%)	
**Type of occupation**			>0.9
Veterinarian	10 (66.7%)	5 (33.3%)	
Poulterer	140 (69.0%)	63 (31.0%)	
Poultry technician	31 (68.9%)	14 (31.1%)	
Manager	38 (66.7%)	19 (33.3%)	
Support functions	4 (66.7%)	2 (33.3%)	
**Farm status**			0.12
Infected farm	65 (62.5%)	39 (37.5%)	
Uninfected farm	158 (71.2%)	64 (28.8%)	

**Fig 3:**
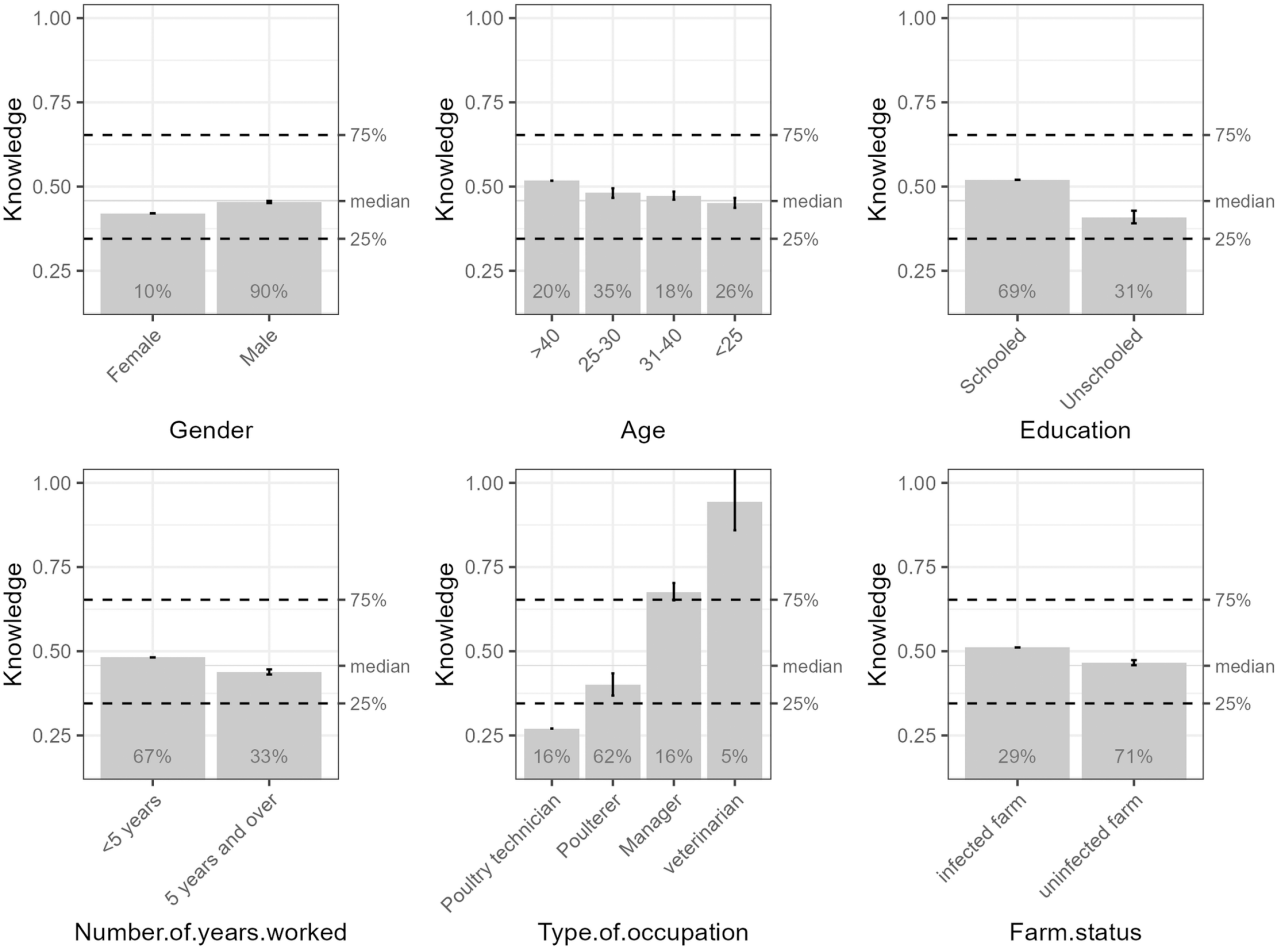
Cumulative local effects on the knowledge of poultry farm personnel in Coyah and Forecariah.

The graphs in Fig 3 visualise the differences and trends in the accumulation of local effects of farm workers’ socio-demographic and occupational characteristics on their knowledge of avian influenza. Age, education and type of occupation are significant factors in this knowledge. Workers over 40 years had a higher cumulative effect than other age groups, indicating they had more knowledge of the disease than other age groups. Educated people have a differentiated knowledge from the uneducated. Veterinarians and farm managers showed a higher cumulative effect than other poultry workers.

**Fig 4:**
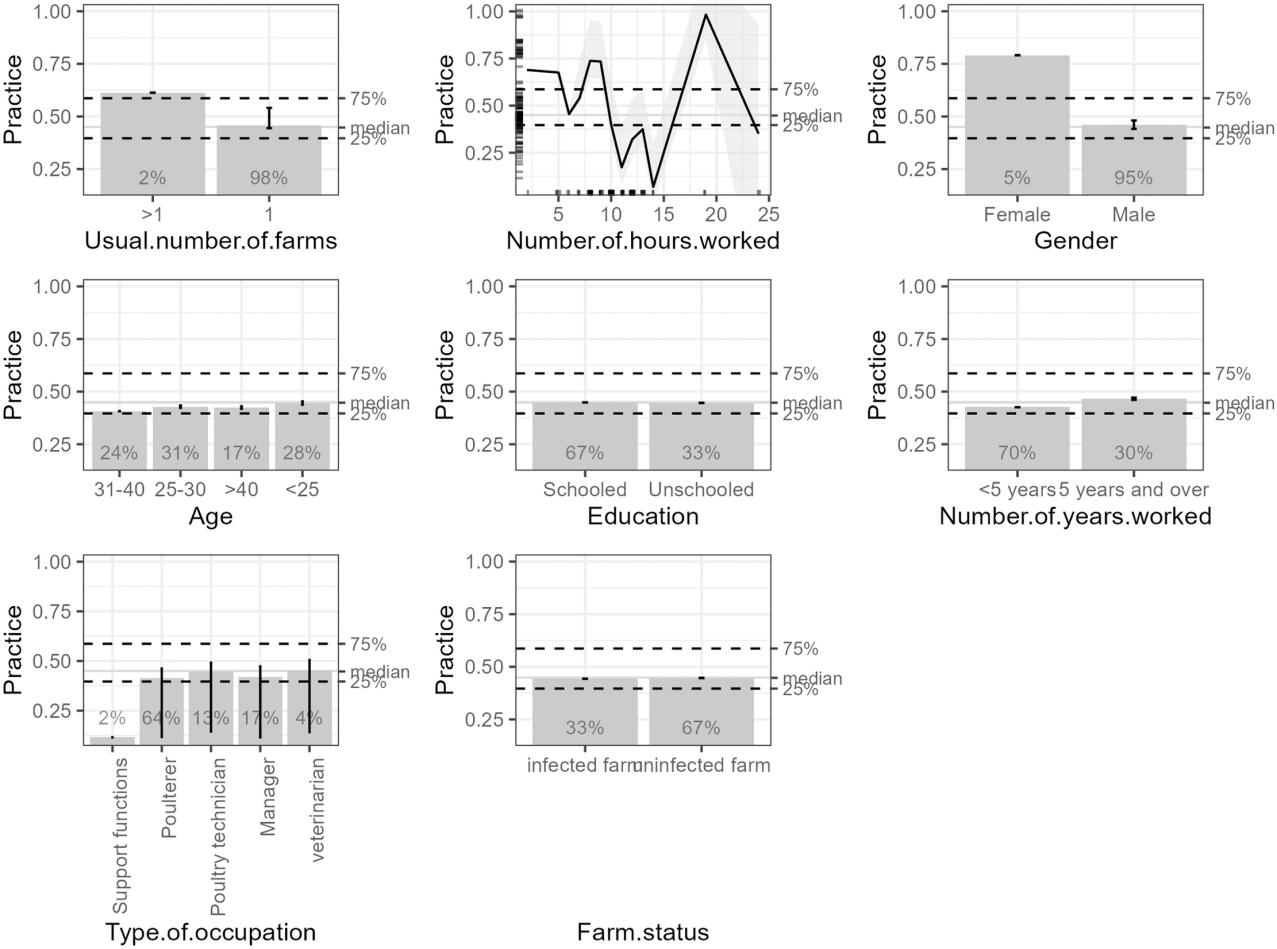
Cumulative local effects on the practices of poultry farm personnel in Coyah and Forecariah.

The graphs in Fig 4 present information on the determinants of poultry workers’ understanding of the definition and modes of transmission, the groups of professionals at risk, and avian influenza prevention measures based on their socio-demographic and professional characteristics. The analysis indicated that these workers’ significant determinants of good practices were the number of farms managed, the number of working hours, and gender. Thus, participants managing multiple farms exhibited different practices than those working on a single farm. However, a strong non-linear association was observed between the number of hours worked and farming practices. Overall, it should be noted that as the number of hours worked increased, fewer good practices were observed. Similarly, gender differences reveal that compliance with practices is higher among women than men.

## Discussion

This study examined the knowledge and practices related to avian influenza among poultry farm workers in the rural areas of Guinea. Conducted in a non-epidemic context, it aimed to raise workers’ awareness of zoonotic diseases such as avian influenza, emphasizing their danger to both animal and human health. The aim is for these results to be considered in designing interventions aimed at improving their knowledge and practices to prepare them better to deal with the emergence and re-emergence of zoonotic diseases such as avian flu. To our knowledge, this study is the first to evaluate the knowledge and practices of poultry farm workers regarding avian influenza in Guinea.

The results of this study indicated that more than half of the respondents had heard about avian influenza several years before the recent outbreak. Their primary sources of information were health workers, friends, fellow farmers, and employers. This can be attributed to the effective collaboration between animal health officials and the managers and owners of various farms, facilitated by an information and communication platform established for this purpose since the onset of the epizootic. Additionally, some farms in these prefectures are located very close. A study in Indonesia also revealed that more than two-thirds (67.0%) of respondents had heard of avian infection. Their main sources of information included health workers (36.0%) and the media, particularly television (34.0%). Several other studies have identified television and radio as the primary sources of information [20–24]. In a previous study, greater knowledge was observed among those whose information sources were health professionals and employers [17].

In this study, the overall level of knowledge was relatively low. The workers demonstrated a limited understanding of avian influenza, particularly regarding its definition and modes of transmission; however, the majority exhibited a good awareness of the occupational groups at risk of contracting the virus and the preventive measures. This may be attributed, on one hand, to their being confronted with this epizootic for the first time and, on the other hand, to the fact that most of these workers had less experience in this field.

Educated workers demonstrated a good understanding of the definition, modes of transmission, occupational groups at risk, and preventive measures. This finding aligns with a study conducted in Indonesia, which reported that the majority of participants had a solid understanding of avian infection as a contagious disease transmissible from birds to other birds, animals, or humans [25]. Previous studies of poultry farmers in Italy, Nigeria, and China found that knowledge of HPAI was significantly higher among those with greater educational attainment and those perceived to be more susceptible to the infection [26–29].

Regarding the workers’ occupation on farms, poultry farmers and technicians exhibited lower knowledge of the definition and modes of transmission compared to other professionals, whereas veterinarians and managers demonstrated a good understanding of the occupational groups at risk (p < 0.001) and prevention measures. This discrepancy was likely due to their greater awareness of poultry diseases in general and avian influenza in particular, partly resulting from their higher level of education. However, the gaps in knowledge are worrying and represent a limitation in disease management in these localities. Future campaigns should, therefore, focus specifically on raising awareness and training all poultry workers. Regarding workers’ behaviour, regardless of their profession or role on the farm, poor practices were observed among most workers, particularly the younger ones. This may be attributed to their limited experience in the poultry industry. Conversely, those who worked on farms previously infected with avian influenza had a higher good practice score (37.5%) than those on non-infected farms (28.8%). This could be explained by the fact that the former had already faced this epizootic, which caused significant economic losses, making them more aware of the importance of adopting good safety practices.

This study has several limitations. Firstly, it could not be conducted on all the poultry farms listed by the national veterinary services, not only due to the impact of the previous epizootic, which led to the closure of several farms in these two prefectures but also because some farmers were reluctant to participate in the study. Furthermore, our participants were not questioned about the reasons for their attitudes toward avian influenza or their vaccination of avian flu status. Additionally, no objective observations, such as direct observations, were collected to confirm self-reported behaviour. Consequently, all these limitations could be addressed in future studies.

## Conclusion

The study revealed a low level of knowledge and poor practices among poultry farm workers despite the outbreak of the avian influenza epizootic. The high demand for additional information on avian influenza evoked by these workers should be considered in the design of interventions targeting high-risk workers to improve their knowledge and practices and better prepare them to cope with the emergence and re-emergence of zoonoses such as avian influenza.

## Supporting information

S1 Data The Excel spreadsheet contains, in separate sheets, data on respondents’ characteristics, knowledge and practices, as well as data for Figures 2, 3 and 4. (XLSX)
